# Behavioral and neurophysiological evidence for increased cognitive flexibility in late childhood

**DOI:** 10.1038/srep28954

**Published:** 2016-06-28

**Authors:** Nicole Wolff, Veit Roessner, Christian Beste

**Affiliations:** 1 Cognitive Neurophysiology, Department of Child and Adolescent Psychiatry, Faculty of Medicine of the TU Dresden, Germany; 2 Experimental Neurobiology, National Institute of Mental Health, Klecany, Czech Republic

## Abstract

Executive functions, like the capacity to control and organize thoughts and behavior, develop from childhood to young adulthood. Although task switching and working memory processes are known to undergo strong developmental changes from childhood to adulthood, it is currently unknown how task switching processes are modulated between childhood and adulthood given that working memory processes are central to task switching. The aim of the current study is therefore to examine this question using a combined cue- and memory-based task switching paradigm in children (N = 25) and young adults (N = 25) in combination with neurophysiological (EEG) methods. We obtained an unexpected paradoxical effect suggesting that memory-based task switching is better in late childhood than in young adulthood. No group differences were observed in cue-based task switching. The neurophysiological data suggest that this effect is not due to altered attentional selection (P1, N1) or processes related to the updating, organization, and implementation of the new task-set (P3). Instead, alterations were found in the resolution of task-set conflict and the selection of an appropriate response (N2) when a task has to be switched. Our observation contrasts findings showing that cognitive control mechanisms reach their optimal functioning in early adulthood.

Executive functions, like the capacity to control and organize thoughts and behavior, develop from childhood to young adulthood and are associated with prefrontal cortex (PFC) maturation. One important aspect of executive functions is cognitive flexibility, which represents the ability to adapt behavior in response to changing environmental conditions^1^,^2^. Cognitive processes related to switching and cognitive flexibility have been investigated intensively^3^,^4^. It is well known that task switches usually require more time and are performed less accurately than task repetitions. These trade-offs are known as “switch costs”^3^,^4^. Since these abilities require the involvement of the PFC^5^,^6^,^7^ and cortico-striatal networks^8^,^9^,^10^,^11^, which crucially develop during the course of the first two decades of life^12^, it is commonly suggested that executive functions reach their peak in early adulthood^13^. In contrast to this it was also observed that children, starting at as early as 6–7 years^14^, 10–11 years^15^ or from 15 years^16^ on show similar overall performance compared to young adults in several aspects of cognitive flexibility, e.g. during rule switching within the Wisconsin Card Sorting Test^15^,^17^, during a figure matching paradigm^14^ or during a cued selective attention task^18^.

It has therefore been suggested that set switching may be associated with subdivisions of the PFC that underlie diverging maturational processes^15^. Moreover, these dissociable subprocesses of cognitive flexibility seem to show specific and independent developmental trajectories^15^,^19^. Task switching and cognitive flexibility are usually examined with paradigms using visual cues to signal rule changes. However, task switching may also be triggered from a memory domain^4^,^20^, meaning that subjects have to remember the rule as well as the time point of rule changes. Memory-based switching thus increases working memory demands and complicates task switching^20^. This is of particular relevance in developmental contexts, since working memory processes underlie strong changes from childhood to adulthood^21^.Specifically, working memory capacity continuously increases^22^,^23^, reaching adult level at around the age of 15^16^.

Although task switching and working memory processes are known to undergo strong developmental changes from childhood to adulthood, it is currently unknown how task switching processes are modulated between childhood and adulthood given that working memory processes are central to task switching. The aim of the current study is therefore to examine this question using neurophysiological (EEG) methods. The use of EEG methods makes it possible to distinguish underlying neural subprocesses of early attentional selection from later response selection stages^20^ by examining distinctive event-related potential components (ERPs). It is thus possible to examine which neural subprocesses in the information processing cascade underlie possible differential behavioral effects of cue-based and memory-based task switching between late childhood and adulthood. It is most likely that differences between children and young adults are stronger during memory based task switching, compared to cue-based task switching. This is because both task switching and working memory processes are immature in children compared to adults.

On a neurophysiological level it has been shown that ERPs reflecting response selection, like the negative-going fronto-central ERP component N2, as well as the subsequent positive-going parietal-central ERP component P3, which also reflects updating of working memory processes are particularly important cognitive subprocesses during task switching^24^,^25^,^26^. The N2 has for example been interpreted as an index of the resolution of task-set conflict and the selection of an appropriate response when a task has to be switched (refs 27, 28, 29). Developmental research showed the N2 to be decreased in amplitude with increasing age, comparing both children with adolescents^30^ and children with adults^31^,^32^. This has been suggested to reflect the development of cognitive control^30^. It can thus be assumed that i) children as compared to young adults show a generally increased N2 amplitude and ii) that processes which more strongly rely on cognitive control may elicit an increased N2 as compared to processes in which less cognitive control is involved. The P3 has been interpreted as reflecting the updating, organization, and implementation of the new task-set^25^,^28^ and is increased in cue- as compared to memory-based task switching. With respect to developmental studies, the P3 has been found to be decreased in latency as well as to be differentially distributed (e.g. being larger at parietal sites and absent at fronto-central sites) in children (9–10 years) as compared to young adults^32^. These observations have been suggested to reflect the recruitment of additional and/or different updating-related cognitive processes in children as compared to adults^31^,^32^. Accordingly, if updating processes are relevant for cue and/or memory based switching, it can be assumed that underlying correlates of these processes differ slightly between children and young adults. Finally, task switching seem to reflect the re-adjustment of internal task representations^4^ which is accompanied by continuous processes of reconciliation and monitoring in order to activate new and to inhibit former task sets. Therefore, we assume that early ERP components, like the positive-going P1 and the negative-going N1, both maximal over the parietal scalp and known to be sensitive for processes like sensory gating (P1) or early attentional selection (N1)^33^,^34^,^35^,^36^ may not be affected during cue- and memory based task switching^20^. We examined these study questions in an established task switching paradigm^27^ involving cue- and memory-based task switching blocks performed by children and young adults while ERPs were being recorded. In sum, since switching processes are associated with diverging maturation processes of the PFC^15^, we assume decreased performance in children as compared to young adults in both cue- and memory-based blocks. With respect to neural correlates, we assume for behavioral effects to be reflected by neural subprocesses. We focus on ERPs that are related to processes of early selective attention (P1 and N1) as well as on response selection (N2) and updating (P3), assuming that response selection processes (N2) best reflect behavioral performance.

## Results

### Behavioral Results

#### Switch costs

Switch costs (SC) were obtained for correct trials by subtracting response times (RTs) during task repetition from RTs during task switching (cue-based SC = RT cue-based-switching – RT cue-based-repetition; memory-based SC = RT memory-based-switching – RT memory-based-repetition).

**Figure 1:**
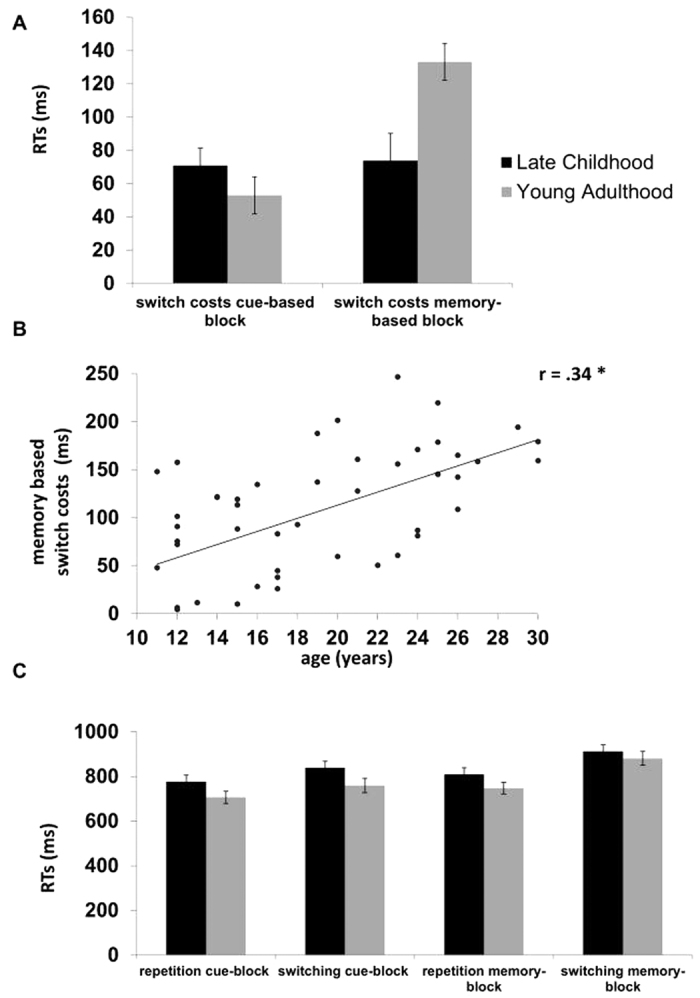
Behavioral Data. **(A)** Shows switch costs, comparing children and young adults. **(B)** Scatter plots showing the correlation between memory-based switch costs (RT) and participant age. The asterisk represent a significant correlation (*p* = 0.009). **(C)** Shows RTs for all blocks (cue- and memory-based) and conditions (switching and repetition) comparing late childhood and young adulthood. Error bars depict standard error of the mean (SEM).

A mixed effects ANOVA on switch costs using the within-subject factor “block (cue- vs. memory-based)” and the between subject factor “group (children vs. young adults)” revealed a main effect “block” (*F*[1,48] = 13.09 *p *< 0.001, *η*^*2*^ = 0.21), indicating lower switch costs in the cue-based (62 ms ± 8), as compared to the memory-based block (104 ms ±10). These effects were further specified by the significant interaction of “block x group” (*F*[1,48] = 11.28 *p* < 0.002, *η*^*2*^ = 0.19, refer Fig. 1A).

Post hoc t-tests, analyzing both groups separately, revealed significant differences in switch costs between the two conditions in young adults (*t*[24] = 6.27, *p* < 0.001), indicating significantly higher switch costs during the memory-based block (133 ms ±11) as compared to the cue-based block (53 ms ± 13). In contrast, children showed no significant effect (*p *= 0.877), indicating comparable switch costs between the memory- (74 ms ± 16) and the cue-based block (71 ms ± 10). Post hoc t-tests, analyzing between group effects, revealed significant switch costs differences between groups in the memory-based block (*t*[48] = −3.01, *p* = 0.004), indicating significantly reduced switch costs in children (74 ms ± 16) as compared to young adults (133 ms ± 11). In contrast switch costs in the cue-based block did not differ significantly (*p *> 0.2) between children (71 ms ± 10) and young adults (53 ms ± 11).

Supporting these results further, we observed a significant positive correlation between memory-based switch costs and age (*r *= 0.34, *p *= 0.009), indicating significantly increased switch costs with increasing age. In contrast no significant correlation (*p *> 0.3) was observed for age and switch costs during the cue-based condition (refer Fig. 1B). Finally, we tested whether switch-costs during cue- and switch-costs during memory-based conditions are different, by testing both against each other. We obtained no significant effect (*p* = 0.76), indicating that the effects are different from each other.

#### RTs

In addition to the difference measure “switch costs” we analyzed single RTs for each block and condition separately to examine whether the obtained group effects are due to RT differences on switch or on repetition trials. The mixed effects ANOVA on mean RTs with the within-subject factors “condition” and “block” and the between subject factor “group”, yielded a significant main effect of “block” (*F*[1,48] = 16.77, *p* < 0.001, *η*^*2*^ = 0.259), indicating faster RTs during the cue- (808 ms ± 20.16) versus the memory-based block (861 ms ± 19.18). Further, the main effect of “condition” was also significant (*F*[1,48]* *= 152.65, *p* < 0.001, *η*^*2*^ = 0.761), indicating faster RTs during task repetition (793 ms ± 18.01) versus task switching (876 ms ± 19.69). The main effect of the between-subjects factor “group” also reached significance (*F*[1,48]* *= 10.70, *p* = 0.002, *η*^*2*^ = 0.182), demonstrating faster RTs in adults (774 ms ± 31.53) compared to children (895 ms ± 29.66). While the interaction “condition x block” failed to reach significance (*F*[1,48]* *= 2.42, *p* = 0.126, *η*^*2*^ = 0.048), we observed significant interactions between “condition x group” (*F*[1,48]* *= 4.61, *p* = 0.037, *η*^*2*^ = 0.088) as well as “condition x block x group” (F[1,48]* *= 11.28 *p* = 0.002, *η*^*2*^ = 0.190) (refer Fig. 1C).

Post hoc tests, analyzing both groups separately, revealed the following pattern: Young adults showed significant effects of “condition” (*F*[1,24]* *= 106.04, *p* < 0.001, *η*^*2*^ = 0.815), and “block” (*F*[1,24]* *= 18.37, *p* < 0.001, *η*^*2*^ = 0.433) which were further specified by the significant interaction “condition x block” (*F*[1,24]* *= 39.27, *p* < 0.001, *η*^*2*^ = 0.621). This shows significantly higher RTs during task switching in the memory-based (881 ms ± 33) as compared to the cue-based (759 ms ± 33) block, as well as significantly higher RTs during task switching (881 ms ± 33) as compared to task repetition (748 ms ± 31) in the memory-based block.

In contrast, children, showed only a significant effect of “condition” (*F*[1,24]* *= 53.62 *p* < 0.001, *η*^*2*^ = 0.691), indicating higher RTs during task switching (931 ms ± 31) versus task repetition (859 ms ± 28). Neither “block” (*F*[1,24]* *= 2.02 *p *= 0.168, *η*^*2*^ = 0.078) nor the interaction between “block and condition” (*F*[1,24]* *= 0.024, *p *= 0.877, *η*^*2*^ = 0.001) revealed significant results. Details on the distribution of RT data in both groups including a statistical analysis on these can be found in supplementary analysis 1.

#### Accuracy

A mixed effects ANOVA on accuracy (percentages of hits) using the within-subject factors “condition” and “block” and the between subject factor “group” yielded a significant main effect of “condition” (*F*[1,48]* *= 22.89 *p* < 0.001, *η*^*2*^ = 0.323), indicating higher accuracy during task repetition (93.02% ± 0.89) than during task switching (90.73% ± 0.98). No other effects were significant (p > 0.079).

### Neurophysiological Results

#### Attentional ERPs

**Figure 2:**
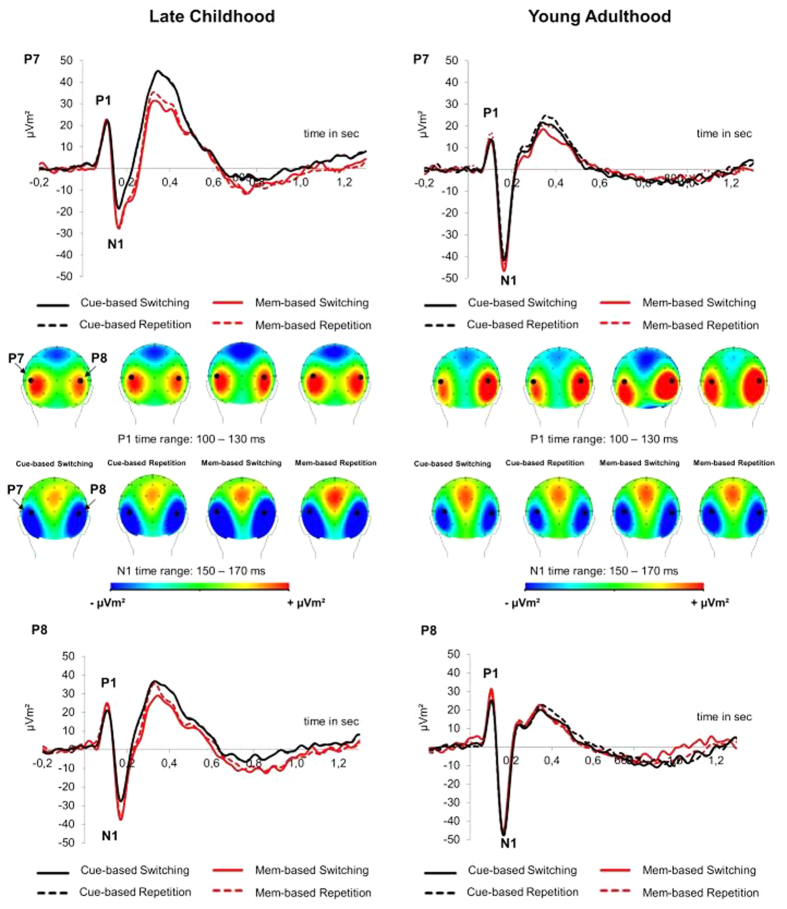
Attentional ERPs. Grand average event-related potentials (ERPs) and voltage maps at occipito-temporal electrodes (P7, P8), depicting P1 and N1 components for the two blocks (cue- and memory-based) and conditions (switching and repetition) in late childhood (left) and young adulthood (right).

Target-locked (i.e. time-locked to the stimulus to which a response was required) ERPs were analysed by calculating mixed-effects ANOVAs using the within-subject factors “electrode” (P7, P8), “condition” and “block”, as well as the between-subjects factor “group”. The P1 and N1 ERPs are shown in Fig. 2.

#### P1

 The mixed effects ANOVA on the P1 amplitudes revealed a significant main effect of block (*F*[1,48]* *= 5.47 *p *= 0.024, *η*^*2*^ = 0.102), indicating increased P1 amplitudes in memory- (21.36 μV/m^2^ ± 2.95) as compared to cue-based blocks (18.85 μV/m^2^ ± 2.64). Moreover, the interactions “electrode x group” was significant (*F*[1,48]* *= 4.24 *p *= 0.045, *η*^*2*^ = 0.081), indicating increased P1 amplitudes in children as compared to young adults at electrode P7 (25.70 μV/m^2^ ± 3.85 vs 12.04 μV/m^2^ ± 3.85) versus electrode P8 (22.33 μV/m^2^ ± 4.85 vs 20.36 μV/m^2^ ± 4.85). The interaction “electrode x block” (*F*[1,48]* *= 7.25 *p *= 0.010, *η*^*2*^ = 0.131), indicated increased P1 amplitudes during memory as compared to cue-based blocks at electrode P8 (23.37 μV/m^2^ ± 3.72 vs 19.33 μV/m^2^ ± 3.25) versus electrode P7 (19.36 μV/m^2^ ± 2.87 vs 18.38 μV/m^2^ ± 2.68). Finally, we observed a significant interaction of “block x condition x electrode” (*F*[1,48]* *= 5.36 *p *= 0.025, *η*^*2*^ = 0.100). Post hoc tests used to analyze both electrodes separately revealed a significant interaction of “block x condition” at electrode P7 (*F*[1,48]* *= 5.21 *p *= 0.027, *η*^*2*^ = 0.098). This shows increased P1 amplitudes during task switching (18.86 μV/m^2^ ± 2.76) versus task repetition (17.90 μV/m^2^ ± 2.68) in the cue-based block. A reversed pattern, namely increased P1 amplitudes during task repetition (20.59 μV/m^2^ ± 2.87) versus task switching (18.12 μV/m^2^ ± 2.96) was found in the memory-based block. Post hoc tests analyzing electrode P8 revealed no significant interaction between “block x condition” (*F* < 1).

#### N1

 The mixed-effects ANOVA on N1 revealed a significant effect of block (*F*[1,48]* *= 5.81, *p *= 0.020, *η*^*2*^ = 0.108), indicating increased N1 amplitudes (−30.00 μV/m^2^ ± 3.54) in memory- as compared to cue-based blocks (−27.22 μV/m^2^ ± 3.61). The effect was further specified by the significant interaction of “block x electrode” (*F*[1,48]* *= 5.49 *p *= 0.023, *η*^*2*^ = 0.103), showing that the N1 amplitude differences between cue- and memory based blocks were stronger at electrode P7 (−23.83 μV/m^2^ ± 3.60 vs −8.22 μV/m^2^ ± 3.76) than at electrode P8 (−0.61 μV/m^2^ ± 4.15 vs −1.78 μV/m^2^ ± 3.98). In addition, the analysis revealed a significant effect of group (*F*[1,48]* *= 7.95 *p *= 0.0074, *η*^*2*^ = 0.142), indicating generally increased N1 amplitudes in young adults (−8.55 μV/m^2^ ± 4.99) as compared to children (−8.67 μV/m^2^ ± 4.99).

#### Response-selection ERPs

**Figure 3:**
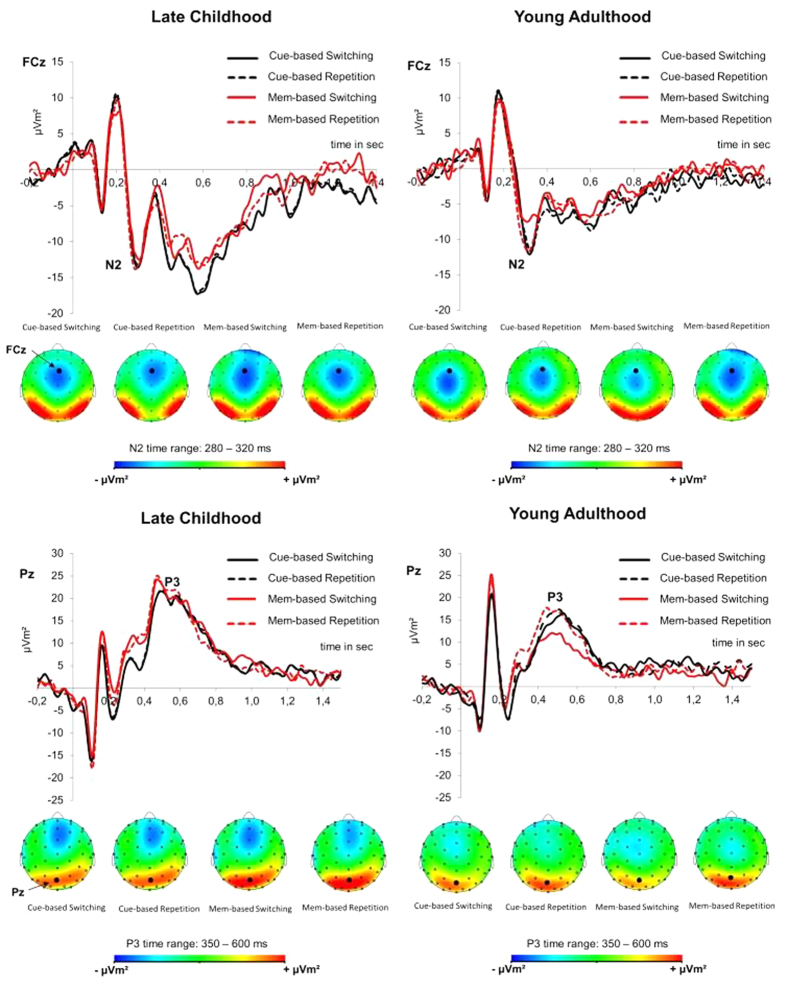
Response-selection ERPs. Grand average event-related potentials (ERPs) and voltage maps at the central electrode (FCz) showing the N2 and at the parietal central electrode (Pz) showing the P3, for the two blocks (cue- and memory-based) and the two conditions (switching and repetition) in late childhood (left) and young adulthood (right).

Target-locked ERPs reflecting response selection processes (i.e., N2 and P3) were analysed by calculating mixed-model ANOVAs with the within-subject factors “condition” and “block”, as well as the between-subjects factor “group”. The ERPs are shown in Fig. 3

*N2*: A mixed-effects ANOVA on N2 revealed a significant main effect of “block” (*F*[1,48]* *= 6.52 *p *= 0.014, *η*^*2*^ = 0.120), which was further specified by the significant interaction of “condition x block” (*F*[1,48]* *= 5.04 *p *= 0.029, *η*^*2*^ = 0.095), indicating significantly increased N2 amplitudes (*t*[49]* *= −0.82, *p *= 0.007) during memory-based task repetition (−3.21 μV/m^2^ ± 1.37) as compared to memory-based task switching (−0.55 μV/m^2^ ± 1.39) and no corresponding effect in the cue-based condition (*p *= 0.69). This interaction was further specified by the significant interaction of “condition x block x group” (*F*[1,48]* *= 4.12 *p *= 0.049, *η*^*2*^ = 0.079). Post Hoc tests analyzing both groups separately indicate a significant interaction of “condition x block” in young adults (*F*[1,24]* *= 17.47 *p* > 0.001, *η*^*2*^ = 0.421), demonstrating significantly higher N2 amplitudes (*t*[24]* *= 3.75, *p* > 0.001) during memory-based repetition (−2.34 μV/m^2^ ± 1.27) versus memory-based switching (−0.83 μV/m^2^ ± 1.58) and no corresponding effect in the cue-based condition (*p *= 0.41). In contrast, in children no significant interaction of “condition x block” was observed (F < 1), indicating no significant differences between cue- and memory based switching and repetition trials (all *p* > 0.05). The N2 effects parallel the behavioral pattern of the observed RT effects. Further details on N2 peaks and latencies can be found in supplementary analysis 4.

*P3*: A mixed-effects ANOVA on P3 revealed a significant main effect of “condition” (*F*[1,48]* *= 35.70 *p *< 0.001, *η*^*2*^ = 0.427), showing increased P3 amplitudes during repetition (16.43 μV/m^2^ ± 2.14) as compared to switching (11.85 μV/m^2^ ± 1.72) trials and a significant main effect of “block” (*F*[1,48]* *= 9.74 *p *= 0.003, *η*^*2*^ = 0.169), showing increased P3 amplitudes during memory- (15.40 μV/m^2^ ± 1.98) vs cue-based (12.88 μV/m^2^ ± 1.90) blocks. These effects were further specified by a significant interaction of “block x condition” (*F*[1,48]* *= 14.92 *p *< 0.001, *η*^*2*^ = 0.237), indicating significant increased amplitudes during repetition vs switching trials during both, memory- (16.32 μV/m^2^ ± 2.19 vs 9.42 μV/m^2^ ± 1.75, *t*[49]* *= 5.88, *p* > 0.001) and cue-based (16.54 μV/m^2^ ± 2.19 vs 14.26 μV/m^2^ ±1.86, *t*[49]* *= 2.83, *p *= 0.006) blocks. Finally a significant interaction of “group x condition” (*F*[1,48]* *= 6.02 *p *= 0.018, *η*^*2*^ = 0.111) was found. Post hoc test analyzing both groups separately revealed significant effects of “condition” in both groups, in young adults (*F*[1,24]* *= 7.99 *p *= 0.009, *η*^*2*^ = 0.250) as well as in children (*F*[1,24]* *= 6.02 *p *= 0.018, *η*^*2*^ = 0.111), indicating increased P3 amplitudes during repetition vs switching trials. However the effect of condition was especially pronounced in young adults (19.83 μV/m^2^ ± 2.35 vs 13.37 μV/m^2^ ± 1.58) as compared to children (13.03 μV/m^2^ ± 3.57 vs 10.33 μV/m^2^ ± 3.57) which can be seen in the larger difference between repetition and switch (6.46 μV/m^2^ vs 2.70 μV/m^2^) trials and the corresponding increased effect size (*η*^*2*^ = 0.250 vs *η*^*2*^ = 0.111) in young adults vs children. No further main effect or interaction was observed (all *p* > 0.14).

## Discussion

We investigated in what way neurophysiological processes underlying cognitive flexibility are affected by age and more specifically to what extent memory- and cue-based task switching are differently modulated between late childhood and young adulthood.

Parts of the behavioral results are well in line with the literature showing increased switch costs as well as generally increased RTs during memory- versus cue-based switching^20^,^34^. In addition we observed lower accuracies during memory- versus cue-based blocks^37^,^38^ as well as during switching versus repetition trials^37^,^38^. However, the results on differences between children and young adults in the memory-based switching block were unexpected, since children performed better in memory-based task switching than young adults and there were no behavioral group differences in the cue-based task switching condition. We observed no group effects or interactions in the accuracy data, indicating that children show the same accuracy as young adults, but are able to perform the task significantly faster. This suggests that any group differences observed do not arise due to task difficulty^39^, but rather stem from development-related differences in the execution of the task. Interestingly and further corroborating the interpretation of a developmental effect, we observed a positive correlation between memory-based switch costs and participant age. This was absent for cue-based switch costs, indicating an increase of switch costs with increasing age under memory load. Accordingly, the behavioral data indicate that cognitive flexibility triggered by working memory processes seems to be highly affected by developmental aspects, while cue-based switching and thus processes of cognitive flexibility without working memory load seem to represent age-independent processes between late childhood and young adulthood^13^. Research on the task switching paradigm in childhood is still limited. However, although results are inconsistent regarding the exact age, most studies indicate similar switch costs in cue-triggered task switching paradigms between children and adults^13^,^16^,^18^,^19^,^37^,^40^, as also found in the current study. These studies show that cue-triggered switch costs decrease significantly in children from 7 to 11 to 15 years^16^ and are similar low between the age of 13–17 years as it is the case in young adults^13^,^14^,^18^,^40^. It has been suggested that theoretical explanations of cognitive mechanisms, like the ability to maintain and manipulate two mental task sets which are responsible for switch costs in adults can also be applied to children^14^. Accordingly, the ability to switch between rules was suggested to reach mature level during late childhood^40^. Interestingly, one other study^41^, solely analyzing children in 5 consecutive age groups from 5 to 13 years, indicated that switch costs and errors decrease in children between 5 and 8 years of age, but are similar in children between 9 and 13 years. Moreover another study^42^ comparing children (mean age 9.4 years) to young (mean age 21.5 years) and older adults (mean age 65.3 years) observed similar switch costs in a cue-triggered switching paradigm in all three age groups. This confirms the assumption that cognitive flexibility measured via cue-based switching seems to represent an executive function which is developed relatively early in childhood and remains stable from childhood to old adulthood^42^.

The neurophysiological data point to the mechanisms underlying the behavioral modulations and differential developmental effects. Neurophysiological parameters known to reflect perceptual or attentional processes (i.e. the P1 and the N1)^43^,^44^ did not show interactions with condition, block and group, suggesting that these processes do not explain the observed behavioral effects. However, we observed an interaction between block, condition and electrode in the P1 in *both* age groups, suggesting that early perceptual processes during stimulus perception across participants seem lateralized to the left hemisphere. With respect to the factors block and condition, we further observed that attention to switch and repetition trials is differently modulated depending on block: with higher attention to switch trials in cue-based blocks and higher attention to repetition trials in memory-based blocks. This modulation points to distinct mechanisms underlying cue- and memory-based blocks.

Moreover, in line with other developmental studies^45^,^46^ we observed a main effect of group for the N1, indicating larger N1 amplitudes in young adults as compared to children. This has been suggested to represent maturational changes from childhood to young adulthood^45^. In addition to the group effect and in line with P1 results, we observed an interaction with block and electrode in N1, indicating increased lateralized attentional allocation processes^47^ during memory- vs cue-based blocks. Both interactions with electrode for P1 and N1 suggest a distinct and age-independent left hemispheric distribution of early attentional and perceptual processes during task switching.

Finally we observed interactions for condition and block as well as for group and condition for the P3, indicating increased P3 amplitudes during repetition versus switching trials, in both groups and in both blocks. This indicates that processes related to the updating, organization, and implementation of a new task-set^27^,^48^,^49^,^50^ are especially pronounced during repetition trials, which indicates a facilitated access of repetitions independent of age or block.

Of particular importance are the findings on the N2. Interactive effects of age group and cue- vs. memory-based task switching are in line with the behavioral data. While there were no N2 modulations between memory- and cue-based conditions in children, in young adults, the N2 amplitude during memory-based repetition was more negative as compared to memory-based switching trials. The findings in young adults are contradictory to the literature, in which it is usually shown that the N2 is stronger during switching than during repetition trials^51^,^52^ (at least when classical cue-based task switching paradigms are applied). During task switching, the N2 has been interpreted as an index of the resolution of task-set conflict and the selection of an appropriate response when a task has to be switched (ref. 53). However, it has been suggested that for these switching processes inhibitory control mechanisms are important^54^,^55^. It is possible that under memory-based task switching these inhibitory control mechanisms cannot fully or effectively unfold because the increased working memory load already demands restricted resources of the central executive. Inefficient inhibitory control mechanisms of a previous task set are known to lead to high switching costs (i.e. compromised cognitive flexibility)^53^,^54^. This is what is reflected in the behavioral data pattern in young adults. Concerning the child group and based on the above interpretation, it seems that the central executive is less strained in children than in young adults during memory-based task switching. This is an unexpected finding, because working memory performance usually peaks between 24 and 35 years of age^56^ and cognitive abilities in general rise steeply from infancy to young adulthood with a peak of cognitive control performance in the late teens and early twenties^12^. These changes are often attributed to the effects of synaptic elimination during development. It has been suggested that the decrease of synaptic density leads to reorganization and development of specialized and more efficient networks^19^. There is a peak of synaptic density during early childhood which is followed by major synapse elimination processes throughout early and mid-adolescence^57^. This process, in combination with the late maturation of the PFC, has been suggested to underlie the finding that internally guided behavior, working memory and organizational skills do not reach full functional capacity until late adolescence, or young adulthood, respectively^12^,^57^. The obtained results are in sharp contrast to these assumptions, which have been supported by different lines of research. Conclusions from the current results have thus to be drawn carefully and require further studies. However, aside from findings which are in line with the synaptic pruning hypothesis, several results from longitudinal MRI studies^58^,^59^ might account for the observed results. It has been observed that cortical gray matter volume increases in childhood and peaks at the age of 11–12 years, followed by a decline during post-adolescence^58^. It has also been shown that cortical gray matter volume was observed to be positively correlated with cognitive flexibility^60^; i.e. the larger the grey matter volume is in prefrontal cortices, the better cognitive flexibility is developed. It may be speculated that similar processes underlie the observed paradoxical finding of increased memory-based task switching in children, compared to young adults. Further, the execution of memory vs cue -based switching might be associated with different subdivisions of the PFC, which in turn may be characterized by diverging maturational trajectories. It cannot be ruled out that a combination of processes, i.e. cognitive flexibility *and* working memory, induces a different dynamic that is currently not covered and explainable by existing theories of the development of cognitive functions from childhood to adulthood.

In summary, we obtained an unexpected, paradoxical effect suggesting that memory-based task switching performance is better in late childhood than young adulthood. No group differences were observed in cue-based task switching. The neurophysiological data suggest that this effect is not due to altered attentional selection or processes related to the updating, organization, and implementation of the new task-set, but to alterations in the resolution of task-set conflict and in the selection of an appropriate response when a task has to be switched. Our observation is in contrast to findings showing that cognitive control mechanisms reach their optimal functioning in early adulthood. Future research should focus on developmental processes of cognitive operations that are a combination of different executive control processes in order to examine in more detail if the combination of processes is related to different developmental trajectories than it is the case for one of these cognitive functions alone.

## Material and Methods

### Participants

A total of n* *= 25 healthy young adults (17 females) (*M *= 23.80 ± 0.66) as well as a group of n* *= 25 healthy children (13 females) (*M *= 13.88 ± 0.40) took part in the experiment. All participants were right-handed, had normal or corrected to normal vision, reported no psychiatric or neurological disorders and were free of medication. Written informed consent was obtained from all participants before the test protocol was conducted. The study was approved by the institutional review board of the medical faculty of the TU Dresden and realized in accordance with the Declaration of Helsinki. Participants performed a cue-based and a memory-based task switching block in a counterbalanced order.

### Task switching paradigm & procedure

**Figure 4:**
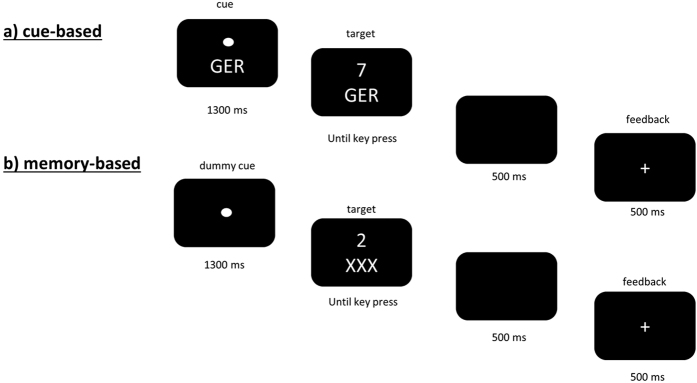
Schematic Illustration of the paradigm. The illustration shows a trial in **(a)** the cue-based condition and **(b)** the memory-based condition.

For stimulus presentation, response recording and EEG triggers “Presentation” software (Neurobehavioral systems, Inc., Version 14.9) was used. Stimuli consisted of digits from 1–9, excluding the number 5, which were presented in white font, centrally on a black computer screen. Digits were either presented in a small or large size (font size 50 vs font size 80) on the screen. The paradigm consisted of two settings: a cue- and a memory-based block. The task was similar to the paradigm applied by Gajewski *et al*.^20^. Here, the task switching paradigm (see Fig. 4) consisted of two sequences: a cue- and a memory-based block. Within the cue-based block, a cue indicates the valid rule out of the following three alternating rules: numeric rule (indicated by the cue “NUM”, as shortcut for the German word “Numerisch”, numeric), parity rule (indicated by the cue “GER”, as shortcut for the German word “Geradzahligkeit”, parity) and font-size rule (indicated by the cue “SCH” as shortcut for the German word “Schriftgröße”, font size). In the numeric rule participants have to decide whether the presented target is greater or less than 5, in the parity rule, whether the presented target is odd or even and in the font-size rule, whether the target is large or small. The rules and thus the cues alternated randomly with an equal distributed probability of cue occurrence, which was set to 33.3% and a frequency of task switching, which was set to 50%. During the memory-based block, participants were told to switch the rule after every third trial out of memory: starting with the numeric task, which needed to be repeated three times. Afterwards, the parity task and then the font-size task were valid, with each task needing to be repeated three times. Then, participants were required to start afresh (e.g. NUM, NUM, NUM, GER, GER, GER, SCH, SCH, SCH, NUM, NUM, NUM, GER; GER, GER, SCH, SCH, SCH, NUM…, refer to Fig. 4). Similar to the cue-base block, the rules had to be applied with an equally distributed probability (33.3%) but with a frequency of task switching, which was set to 33.3%. During both blocks, participants were allowed to take a self-paced period of rest every 33 trials.

Participants were seated in front of a computer screen and performed one practice block (18 trials per block) and 2 experimental blocks (198 trials per block). Participants were instructed to answer as quickly and accurately as possible via button presses with their left index finger (depending on experimental version e.g. if the digit was smaller than five, had small font size or was uneven), or the right index finger (depending on experimental version e.g. if the digit was larger than five, had a large small size or was even). Response key assignment and the sequence of blocks were counterbalanced across participants.

Each block included an equal number of stimuli (digits) as well as responses. Trials were structured as follows: first, a fixation point in combination with the cue stimulus was presented for 1300 milliseconds (ms). The cue-stimulus remained visible until the digit (target) was presented. The digit appeared at the same position at which the fixation point was presented before. A response had to be given within 2500 ms after target-onset. If no response was given within the 2500 ms, the trial was rated as missed response. 500 ms after the response, a feedback was displayed for 500 ms, which was either a plus (in case of a correct response) or a minus sign (after a wrong response). After the feedback the next cue was shown. The response-cue interval (RCI) was set to ~1500 ms and included the response-feedback delay (500 ms), the feedback (500 ms) and the feedback-cue delay jittered between 400 and 600 ms (mean 500 ms). When during the memory-based block three consecutive errors were made, cues were consecutively presented for the next 3 trials helping participants to remember the correct order. Supplementary analysis 2 provides details on how often this occurred for each condition and group. A test session took about 1 hour including arrival, EEG preparation, task explanation, obtaining consent from both parents and children and task performance. Children and young adults received a financial compensation of 10,-EUR.

### EEG recording and analysis

We recorded electroencephalography (EEG) using a 60-channel system (containing the following channels: Cz, FCz, FC1, CP1, Fz, F1, FC3, C3, CP3, P1, AFz, AF3, F5, FC5, C5, CP5, P3, PO1, FP1, FT7, T7, TP7, P7, O1, FT9, TP9, P9, O9, P11, FC2, CP2, CPz, F2, FC4, C4, CP4, P2, Pz, AF4, F6, FC6, C6, Cp6, P4, PO2, Fp2, AF8, FT8, T8, TP8, P8, O2, Oz, FT10, TP10, P10, O10, Iz, P12, AF7). EEG recordings were performed using the Brain Vision Recorder software (Brain Products) with a sampling rate of 500 Hz and electrode impedances under 5 kΩ. Ag/AgCl-electrodes were mounted in an elastic cap and were arranged in equidistant positions. Data analysis was performed using the Brain Vision Analyzer 2 software package. After recording, data were down-sampled to 256 Hz and filtered (band-pass filter from 0.5 to 20 Hz, with a slope of 48 dB/oct each). Raw data were inspected manually in order to reject non-ocular artifacts from the EEG. Afterwards, an independent component analysis (ICA; infomax algorithm) was conducted on the un-epoched data sets in order to remove recurring artifacts. ICA components revealing horizontal and vertical eye movements, blinks and pulse artifacts were manually chosen to be discarded. Afterwards, EEG data was segmented for switch and repeat trials and separately for cue- and memory-based blocks. Only trials with correct responses were analyzed further. The segments started 200 ms before target presentation of the respective trial and ended 1500 ms after its onset. Subsequently, an automated artifact rejection procedure was conducted for all segments. An activity below 0.5 μV in a 100 ms period and a maximal value difference of 200 μV in a 200 ms interval were used as rejection criteria. To eliminate the reference potential from the data and to re-reference the data, we applied a current source density (CSD) transformation^61^ which results in values for amplitudes in μV/m^2^. A baseline correction from −200 ms to −1800 ms prior to target onset was applied; i. e, the baseline was set before the cue onset. Information about the number of trials included in the data analysis after artifact rejection and rejection of incorrect trials can be found in supplementary analysis 3. For statistical analysis we used SPSS (IBM SPSS Statistics, Version 23). In addition, post-hoc tests were Bonferroni-corrected and data were checked for the presence of normal distribution (Kolmogorov Smirnov test, all *p *> 0.05) as well as for the presence of variance homogeneity (Levene test, all *p *> 0.05). We analyzed ERP components at the electrodes of their respective maximum amplitudes relative to pre-stimulus baseline. Since it has been observed that that the topographic distribution of the visual P1 and N1 indicate specific lateralized generators in the occipitotemporal gyrus^33^, mean amplitudes were quantified for P1 and N1 at electrodes P7 and P8 (time window P1: 100–130 ms, time window N1: 150–170 ms). Mean amplitudes were also quantified for N2 at fronto-central electrodes (FCz) in the time window from 280 till 320 ms after target onset. Finally, mean P3 amplitudes were calculated at central electrodes (Pz) between 350 and 600 ms. This choice of electrodes was validated using statistical tests as described in^60^. This validation procedure revealed the same electrode locations.

## Additional Information

**How to cite this article**: Wolff, N. *et al*. Behavioral and neurophysiological evidence for increased cognitive flexibility in late childhood. *Sci. Rep.* **6**, 28954; doi: 10.1038/srep28954 (2016).

## Supplementary Material

Supplementary Information
