# Supplementary material for: Behavioral and neurophysiological evidence for increased cognitive flexibility in late childhood
Source: Sci Rep. 2016 Jun 28;6:28954. doi: 10.1038/srep28954 (PMC4923946; doi:10.1038/srep28954)
Supplement: Supplementary Information [file srep28954-s1.doc]

**Supplemental material**

to the article

**Behavioral and neurophysiological evidence for increased cognitive flexibility in late childhood**

Authors: Nicole Wolff, Veit Roessner, Christian Beste

**Supplementary analysis 1**

Since the groups do not differ with respect to standard deviation or standard error of the mean we did no preprocessing of RTs and presented original RTs. However since the observed interaction of condition x block was not significant in the children’s group we did not offer all RTs for the reader. For more information see Table S1 below.

| Group |  | Cue-based Repetition | Cue-based Switching | Mem-based Repetition | Mem-based Switching |
| --- | --- | --- | --- | --- | --- |
| adults | RTs (ms) | 706.84 | 759.71 | 748.04 | 881.25 |
|  | SD | 144.30 | 166.68 | 155.67 | 164.00 |
|  | SEM | 28.86 | 33.34 | 31.13 | 32.80 |
| children | RTs (ms) | 847.36 | 918.10 | 871.28 | 945.01 |
|  | SD | 151.58 | 154.08 | 131.38 | 156.08 |
|  | SEM | 30.32 | 30.82 | 26.28 | 31.22 |

We reanalyzed our data in more detail and checked whether age groups differ with respect to RT distribution. For this analysis we determined individual SD Cutoffs and excluded trials which differ more than 2SDs from the Mean for both groups separately. Applying this cut off, the analysis revealed that in young adults *M* = 15.2 (± 17.74 SD) and in children *M* = 14.36 (±15.08 SD) trials need to be excluded. Paired t-test comparing both age groups revealed no significant difference between groups (*t*[48] = -0.180, *p =* .858). This shows that the distribution of the RTs is comparable between the groups.

**Supplementary analysis 2**

When three consecutive errors were made during the memory-based block, cues were presented for the next three trials. 18 children (72%) and 16 young adults (64%) performed the memory task without getting help through presented cues. Only 7 children and 8 young adults get help trough presented cues during memory based trials (see Table S2 for more information). Chi Square test revealed that children and young adults did not differ in frequency in which cues where presented (*x*^2^ [4] = 2.51, *p* = .641). Since there were only few trials as well as no frequency differences between groups we decided not excluded those data from the overall analysis.

|  | | | | | | | |
| --- | --- | --- | --- | --- | --- | --- | --- |
|  | | Trials cue shown in memory based block | | | | | Total |
|  |  | 0 | 3 | 6 | 9 | 12 |  |
| Children  Young Adults | number | 18 | 4 | 2 | 0 | 1 | 25 |
|  | % | 72% | 16% | 8% | 0% | 4% | 100% |
|  | number | 16 | 6 | 2 | 1 | 0 | 25 |
|  | % | 64% | 24% | 8% | 4% | 0% | 100% |

**Supplementary analysis 3**

During cue-based switching M = 83.85 trials, (±6.49) were included in the data analysis, during cue-based repetition we included M = 86.35 trials, (±6.17), during memory-based switching M = 51.23 trials, (±4.92) and during memory-based repetition we included M = 115.35 trials, (±9.28). We calculated an ANOVA to analyze differences between conditions/blocks and age groups, which revealed a main effect of condition (*F*[1,48] = 3004.03, *p* >.001, *η^2^* = .984), indicating generally more included trials for repetition trials than for switch trials. There was also an interaction of condition x block (*F*[1,48] = 3062.11, *p* >.001, *η^2^* = .985). The interaction indicates more included trials during memory based repetition as compared to both, memory based switching as well as cue-based repetition. Both effects can be explained by the structure of the paradigm in which the number of switching and repetition trials depends on the block (with more repetition trials during memory based as compared to cue based blocks). Finally we observed a main effect of group (*F*[1,48] = 24.95, *p* >.001, *η^2^* = .342), indicating that in the group of young adults more trials (*M* = 88.24 ± 4.04 ) remained in the analysis after artifact rejection and rejection of incorrect trials, as compared to children (*M*=80.09 ± 9.39).

**Supplementary analysis 4**

A mixed-effects ANOVA on N2 peak amplitudes revealed a significant main effect of “block” (*F*[1,48] = 8.3*2 p =* .006, *η^2^* =.162), indicating significantly increased N2 amplitudes during cue-based blocks (-13.84 µV/m^2^ ± 1.58) as compared to memory-based blocks (-10.63 µV/m^2^ ± 1.34). This main effect was further specified by the significant interaction of “condition x block x group” (*F*[1,48] = 4.10 *p =* .049, *η^2^* =.087). Post Hoc tests analyzing both groups separately indicate a significant interaction of “condition x block” in young adults (*F*[1,24] = 8.25 *p =* .008, *η^2^* =.258), demonstrating significantly higher N2 amplitudes (*t*[24] = 3.56, *p* = .002) during memory-based repetition (-12.94 µV/m^2^ ± 1.47) versus memory-based switching (-6.17 µV/m^2^ ± 1.42) and no corresponding effect in the cue-based condition (*p* = .78). In contrast, in children no significant interaction of “condition x block” was observed (F<1), indicating no significant differences between cue- and memory based switching and repetition trials (all *p*>.69). The N2 effects parallel the behavioral pattern of the observed RT effects.

A mixed-effects ANOVA on N2 peak latencies revealed a significant interaction of “block x group” (*F*[1,48] = 6.21 *p =* .016, *η^2^* =.115). Post Hoc tests analyzing both groups separately indicate no significant main effect of “block”, neither in young adults (*p =* .079) nor in children (*p =* .077). No further effect or interaction was observed in the analysis of N2 latency.
